# Strong purifying selection in endogenous retroviruses in the saltwater crocodile (*Crocodylus porosus*) in the Northern Territory of Australia

**DOI:** 10.1186/1759-8753-3-20

**Published:** 2012-12-05

**Authors:** Amanda Yoon-Yee Chong, Sarah Jane Atkinson, Sally Isberg, Jaime Gongora

**Affiliations:** 1 RMC Gunn Building, B19, Faculty of Veterinary Science, University of Sydney, Sydney, NSW, 2006, Australia; 2 Porosus Pty Ltd, PO Box 86, Palmerston, NT, 0831, Australia

**Keywords:** Crocodylia, Endogenous retrovirus, *Crocodylus porosus*

## Abstract

**Background:**

Endogenous retroviruses (ERVs) are remnants of exogenous retroviruses that have integrated into the nuclear DNA of a germ-line cell. Here we present the results of a survey into the ERV complement of *Crocodylus porosus*, the saltwater crocodile, representing 45 individuals from 17 sampling locations in the Northern Territory of Australia. These retroelements were compared with published ERVs from other species of Crocodylia (Crocodilians; alligators, caimans, gharials and crocodiles) as well as representatives from other vertebrates. This study represents one of the first in-depth studies of ERVs within a single reptilian species shedding light on the diversity of ERVs and proliferation mechanisms in crocodilians.

**Results:**

Analyses of the retroviral *pro*-*pol* gene region have corroborated the presence of two major clades of ERVs in *C*. *porosus* and revealed 18 potentially functional fragments out of the 227 recovered that encode intact *pro*-*pol* ORFs. Interestingly, we have identified some patterns of diversification among those ERVs as well as a novel sequence that suggests the presence of an additional retroviral genus in *C*. *porosus*. In addition, considerable diversity but low genetic divergence within one of the *C*. *porosus* ERV lineages was identified.

**Conclusions:**

We propose that the ERV complement of *C*. *porosus* has come about through a combination of recent infections and replication of ancestral ERVs. Strong purifying selection acting on these clades suggests that this activity is recent or still occurring in the genome of this species. The discovery of potentially functional elements is an interesting development that warrants further investigation.

## Background

Endogenous retroviruses (ERVs) are a group of retrotransposons derived from germ-line integrations of exogenous retroviruses and are found in the genomes of most vertebrate taxa [1]. The ERV complement of mammalian taxa has been studied in detail, particularly in humans, primates, model organisms, and to a lesser extent, domestic species [2-4]. However, there is very little information regarding diversity and distribution of retroviruses in lower vertebrates, with the exception of those of the chicken [5]. Research into the diversity of ERVs within these taxa has focused more on specific elements or the distribution of the various ERV classes across species, rather than detailed studies into the ERV complement of a specific species [6-14]. Thus, there is little data on evolution and diversity of ERVs within individual lower vertebrate species, including crocodilians. To address this, we have investigated the distribution and evolution of these retroelements in the saltwater crocodile (*Crocodylus porosus*).

Once integrated into a host genome, ERVs quickly become defective due to selection against the functional retroviruses [15,16]. While these ERVs are mostly non-functional, degenerate ERVs may also retain the capacity to replicate if the necessary regulatory sequences are present and the proteins required for replication are provided by other functional ERVs [15]. Movement and proliferation of ERVs throughout the genome is one of the processes by which multiple related ERV lineages may occur. These lineages may evolve independently within the host genome, to the point that a single genome may contain many thousands of copies of a provirus from a single infection [16,17].

ERV replication within the genome can occur through a number of mechanisms, such as re-infection, retrotransposition and complementation. The likelihood of each of these occurring is dependent on the functionality of the various retroviral domains. For example, re-infection requires that all retroviral genes are functional, and is the method by which retroviruses may infect other host cells [18]. Replication within host cells may occur through retrotransposition or complementation. Retrotransposition occurs when the ERV utilizes its own encoded domains to integrate proviral copies into new locations in the cellular genome. Complementation is where the proteins required for replication are supplied by other ERVs or exogenous retroviruses [4,18].

Exogenous retroviruses and their endogenous counterparts comprise a large and diverse family that can be divided into seven genera: *Alpharetrovirus*, *Betaretrovirus*, *Gammaretrovirus*, *Deltaretrovirus*, *Epsilonretrovirus*, *Lentivirus* and *Spumavirus*. ERV classification into these genera is generally based on similarity to classified exogenous retroviruses [19]. The discovery of a divergent clade of endogenous retroviruses in the Order Crocodylia (families *Alligatoridae* and *Crocodylidae*) [14] suggests that these taxa may harbor hitherto unseen retroviral diversity, and potentially functional novel elements. Subsequent research has identified two clades of crocodilian ERVs (CERVs) [11]. One of these groups, termed CERV1, falls within the *Gammaretrovirus* related ERVs and has only been isolated from species within *Crocodylidae*, while the other, CERV2, forms a separate cluster distinct from other ERVs. This second clade of ERVs has been identified in a number of species within both *Crocodylidae* and *Alligatoridae*. This evidence for recent and ancient ERV insertions in these taxa makes it an ideal candidate for the exploration of ERV evolution, and the diversification and differentiation of ERVs at species level.

There are 23 recognized species within the Order Crocodylia, belonging to nine genera. *Alligatoridae* consists of the genera *Alligator*, *Caiman*, *Paleosuchus* and *Melanosuchus*, while *Crocodylidae* consists of *Crocodylus*, *Osteolaemus*, *Mecistops*, *Tomistoma* and *Gavialis*[20,21]. *C*. *porosus* has the broadest geographical distribution of all crocodilian species with populations in Australia, the indo-pacific region, South-East Asia and up to India [22,23]. It is one of two crocodilian species found in Australia and the only farmed crocodilian species in this country.

Given the current knowledge of the distribution of ERVs in crocodilians, it would be expected that the majority of ERV sequences will be the result of ancient re-infections and retrotransposition. However, sufficient sequence data is not available to assess the evolutionary processes associated with retroviral proliferation within these species. Crocodilian genomes display a significantly lower mutation rate than other vertebrate species [24-27], providing a good opportunity to study the dynamics of rapidly evolving DNA, such as ERVs in a slow mutation rate genome. *C*. *porosus* is an ideal candidate species for these studies given the interest in sequencing its genome [28] and ready access to samples from specimens hatched in commercial farms.

**Figure 1:**
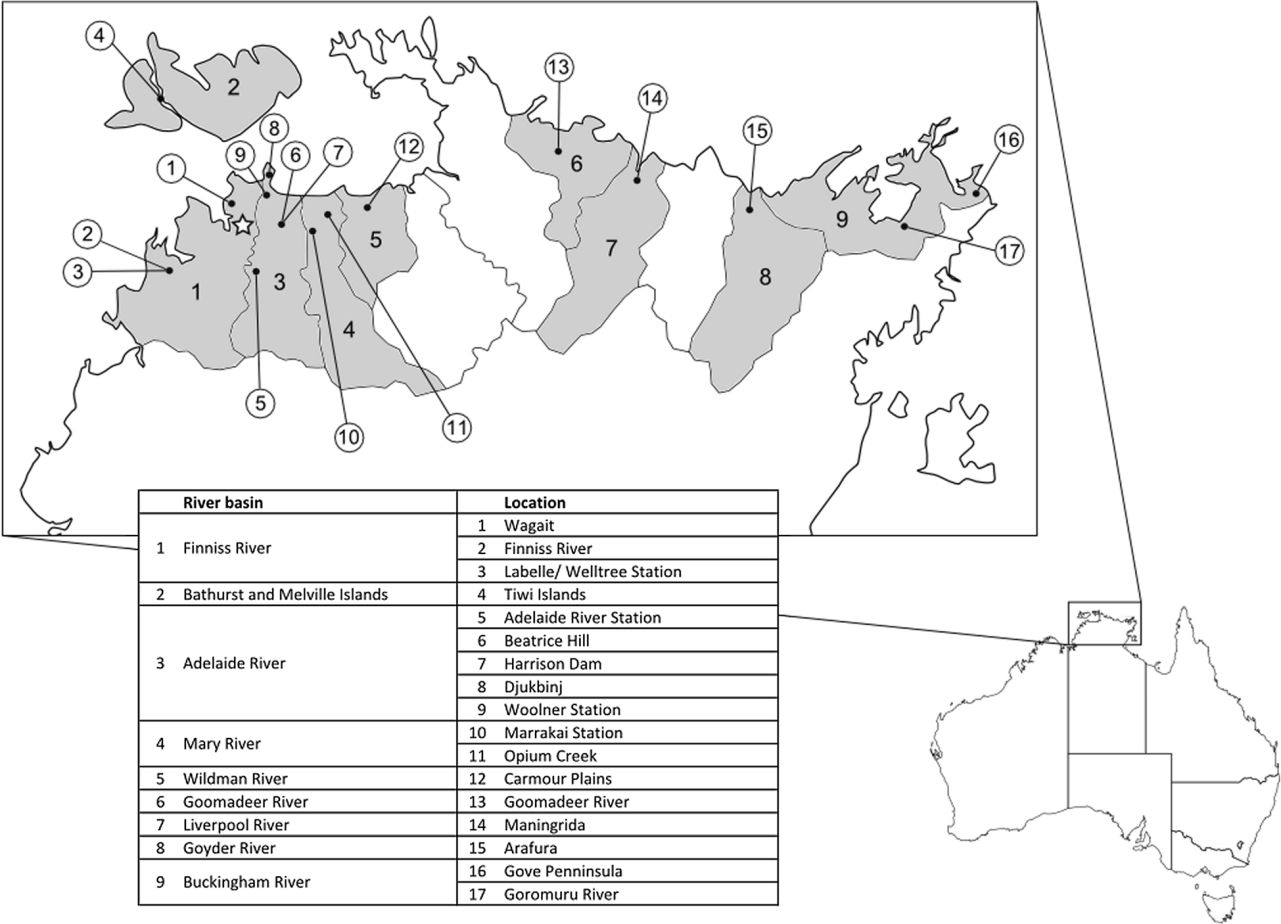
**Sampling locations in the Northern Territory**, **Australia.** Map of the northern end of the Northern Territory, Australia, showing the river basins and locations of sampling sites. Shaded regions indicate the river basins included in this study. Numbers within shaded regions correspond to the basin names in the first column of Table 1, while numbers in circles correspond to the locations listed in the second column. The star indicates the location of Darwin, the largest city in this area. Image adapted from the Australian Bureau of Meteorology (http://www.bom.gov.au).

Here we present the results of a survey into the ERV complement of *C*. *porosus* based on analysis of the *pro*-*pol* gene region. The study focuses on the genetic diversity and potential functionality of these ERV fragments from animals across the Northern Territory of Australia (Figure 1). This is one of the first in-depth studies into the diversity of ERVs within a single reptilian species and will encompass a large number of individuals across a large portion of the range of *C*. *porosus*.

## Results

### Sequence overview

A PCR survey of ERVs in 47 individuals yielded a total of 227 clones, which were subsequently sequenced. These sequences represented 176 novel DNA haplotypes and 126 novel amino acid haplotypes [GenBank: JX157669 to JX157844]. Sequences ranged in length from 665 to 957 nucleotides. Up to 12 unique sequences were identified per individual, with very few sequences coming from more than one clone per individual. All sequences except for two could be assigned to the two CERV clades previously described [11] based on visual inspection and genetic similarity values. A total of 45 haplotypes belonged to clade CERV1 and 129 haplotypes were assigned to clade CERV2. Overall, CERV2 clones were more prevalent across the 17 sampling locations (Figure 1). The proportion of sequences from each of the CERV clades did not appear to vary from the overall average proportion of sequences recovered across all locations (Table 1). Although recent genetic studies have revealed some level of diversity among animals from the same or similar sampling locations [29,30], a comparison with the current dataset was not possible as different regions of the genome (mtDNA and gene coding regions) were used in these studies.

**Table 1:** Sequences obtained from each sampling location and the assigned clades

**River basin**	**Sampling location**	**Number of clutches**	**Number of Individuals**	**Number of sequences**	**CERV1**	**CERV2**	**Other**
Finniss River	Wagait	1	1	3	-	3	-
	Finniss River	2	2	3	-	3	-
	Labelle/Welltree Station	4	4	9	4	5	-
Bathurst and Melville Islands	Tiwi Islands	3	3	10	3	7	-
Adelaide River	Adelaide River Station	2	2	14	1	13	-
	Beatrice Hill	1	2	9	1	7	1
	Harrison Dam	4	4	19	4	15	-
	Djukbinj	4	4	26	6	19	1
	Woolner Station	3	4	12	5	7	-
Mary River	Marrakai Station	5	5	23	4	19	-
	Opium Creek	1	1	2	-	2	-
Wildman River	Carmour Plains	1	1	1	1	-	-
Goomadeer River	Goomadeer River	3	3	21	3	18	-
Liverpool River	Maningrida	2	2	12	3	9	-
Goyder River	Arafura	2	2	4	1	3	-
Buckingham River	Gove Penninsula	3	3	11	3	8	-
	Goromuru River	4	4	19	6	13	-
Total number of samples		45	47	198	45	151	2

BLAST searches and comparisons with sequences in Repbase suggested that one of the outlier sequences, haplotype 58, showed similarity to the exogenous *Epsilonretrovirus*, Walleye Dermal Sarcoma Virus. Haplotype 119 appeared more similar to a gypsy retrotransposon. A third sequence, haplotype 107, also appeared to be divergent from other crocodilian sequences but phylogenetic analysis grouped this within clade CERV2. In addition to this, 18 sequences belonging to clade CERV1 were found to encode intact ORFs (haplotypes 1, 2, 14, 19, 25, 26, 27, 30, 46, 77, 87, 88, 90, 140, 148, 157, 175 and 176). No intact ORFs were identified from CERV2 related ERV fragments. There was no apparent prevalence of intact ORFs from any particular river basin (data not shown).

The YXDD retroviral reverse transcriptase motif was highly conserved in both CERV clades. CERV1 sequences also contained a number of gammaretroviral motifs from the protease and reverse transcriptase domains. CERV2 sequences had regions showing some similarity to spumaviral domains, though only three of the possible six domains were detected (Table 2). Haplotype 58 was shown to contain an *Epsilonretrovirus* motif as well as a number of common gamma- and epsilonretroviral motifs.

**Table 2:** **Conserved retroviral *pro***-***pol *motifs in crocodilian ERV (CERV) sequences**

**CERV clade**	**Motif**	**Genera**	Motif sequence^*^
CERV1	PR2	*Gammaretrovirus*	(A/V)L(V/L)DTG(A/S)TFSM
	PR3	*Gammaretrovirus*	LLG(Q/R)DLLTKL
	RT1	*Gammaretrovirus*	YN(S/T)PILGV(L/P)K(A/V)
	RT2	*Gammaretrovirus*	SVLDLKDAFFSI(P/S(L
	RT3	*Gammaretrovirus*	(Q/R)LMWTVLPQGF(I/V)(A/V)AP
	RT4	*Gammaretrovirus*	LL(H/Q)YVDD(I/L)L
Haplotype 58	PR2	*Gammaretrovirus*	VLLDGTATMSM
	PR3	*Gammaretrovirus*	LLGRDLLCK
	RT1	*Gammaretrovirus*	CNTPVLPVRKP
	RT2	*Epsilonretrovirus*	TVIDLCAAFFPIPV
	RT3	*Gammaretrovirus*	HTLNTQLPQGYTKSP
	RT4	*Gammaretrovirus*	LVQYVDDIL
CERV2	RT2	*Spumavirus*	(A/T)AID(L/P)K(D/E)MF(C/Y)(H/Q)IPL
	RT3	*Spumavirus*	F(E/K)G(C/H/R)VY(E/K)WKVC(P/S)(E/Q)GYKNSP
	RT4	*Spumavirus*	(L/N)SYVDD(I/L)L

^*^Residues presented here are those that occurred in more than one sequence. Motifs are based on those defined by Sperber *et al.*[48]. For the complete alignments, see Additional file 1: Figure S1.

Sequences within each of the CERV clades were highly conserved, with pairwise genetic distances of 0.058 and 0.039 between nucleotide sequences, and 0.071 and 0.084 for amino acid sequences within CERV1 and CERV2 respectively. Visual inspection of the sequence alignments for each of the two clades did not reveal any distinct grouping within CERV1 but suggested that an additional two groups exist within CERV2 (see Additional file 1: Figures S1b and S2b). Within CERV2, genetic distances decreased further when calculated within each of these groups, with distance values of 0.008/0.017 (CERV2a), 0.008/0.011 (CERV2b) and 0.015/0.029 (CERV2c) for nucleotide and amino acid alignments respectively.

The distribution of stop codons and frameshift mutations differed between the two clades. Sequences within CERV1 contained very few stop codons or frameshifts that were shared between sequences, and those that were, tended to be present in only a small number of sequences. In contrast, stop codons and frameshifts within CERV2 sequences were mostly present in all of the sequences within a group.

Recombination analyses detected five recombinant sequences within the two major CERV clades, and two possible recombinants where only trace evidence of a recombination event was detected. Within CERV1, the recombinant sequences were haplotype 1 and *C*. *siamensis* IV, with *C*. *niloticus* I also suspected to be recombinant. Haplotypes 60, 81, and *A*. *mississippiensis* II were detected from clade CERV2, and *A*. *mississippiensis* I was also suspected to be a recombinant sequence (data outlining the boundaries of recombinant regions within each of these sequences and the predicted parental sequences are available in Additional file 1: Table S1). Of these, only haplotypes 1 and 81 within *C*. *porosus* show strong evidence of recombination, being reported as having a significant *P* value using all methods implemented.

The expected parental sequences for recombinant sequences were isolated from different individuals and in some cases from different species. While this reduces the likelihood that the observed recombination occurred during amplification, the possibility cannot be ruled out, since we do not know the full extent of the ERV complement of individuals used in this study. The sites involved in recombination were different in all recombinant sequences detected and did not appear to correspond to specific regions of the *pro*-*pol* domain.

### Selection

Tests for selection across the major clades gave consistent results both across species in Crocodylia and within *C*. *porosus*. Codon based Z-tests suggest that purifying selection is occurring across crocodilian species (CERV1: Z = 7.496, *P* <0.001, CERV2: Z = 2.224, *P* = 0.014), and within *C*. *porosus* (CERV1: Z = 7.060, *P* <0.001, CERV2: Z = 2.633, *P* = 0.005). Comparisons of d_N_/d_S_ across the different ERV clades gave average d_N_/d_S_ ratios under the one ω model between 0.2696 and 0.5539 (Table 3). In all cases, allowing sites to evolve under positive selection produced a better fit in the resulting phylogenies, although the overall d_N_/d_S_ ratios strongly supported purifying selection acting on these elements (the parameters and test statistics are available as Additional file 1: Table S2). Positive selection was detected at a small number of sites in both clades, but these sites do not appear to correspond to retroviral motifs.

**Table 3:** **Average d**_**N**_/**d**_**S**_**for each of the selection scenarios tested**

**Hn**	Model^*^	**Average d**_**N**_**/d**_**S**_	
		**CERV1**	**CERV2**
H0	M0: One ratio	0.4904	0.5539
H1	M3: Discrete	0.5187	0.6898
H2	M1a: Nearly neutral	0.4344	0.3057
H3	M2a: Positive selection	0.5234	0.5864
H4	M7: Beta	0.4217	0.3349
H5	M8: Beta and ω	0.4973	0.6217

^*^Analysis was conducted using PAML [53]. Model names are those defined in the program.

### Sequence clustering and phylogenetic analysis

Nucleotide and amino acid trees created using neighbor joining and maximum likelihood methods present very similar topologies with little phylogenetic differentiation within each clade. Neither neighbor joining nor maximum likelihood methods provided any better resolution of the phylogenetic relationships between the sequences. Overall, the tree topology was similar within both clades, with very short internal and terminal branches (these phylogenetic trees are available as Additional file 1: Figure S2). The lack of phylogenetic resolution is most notable within the clade CERV1. No highly supported groups or lineages were identifiable within *C*. *porosus*, or when these sequences were compared with those of other crocodilian species. Interestingly, we observed a tendency for sequences encoding intact ORFs to cluster within one clade of the CERV1 phylogeny. Within CERV2, phylogenetic trees supported the presence of three groups of sequences within the clade, with moderate bootstrap support, consistent with what was observed with sequence genetic distances.

**Figure 2:**
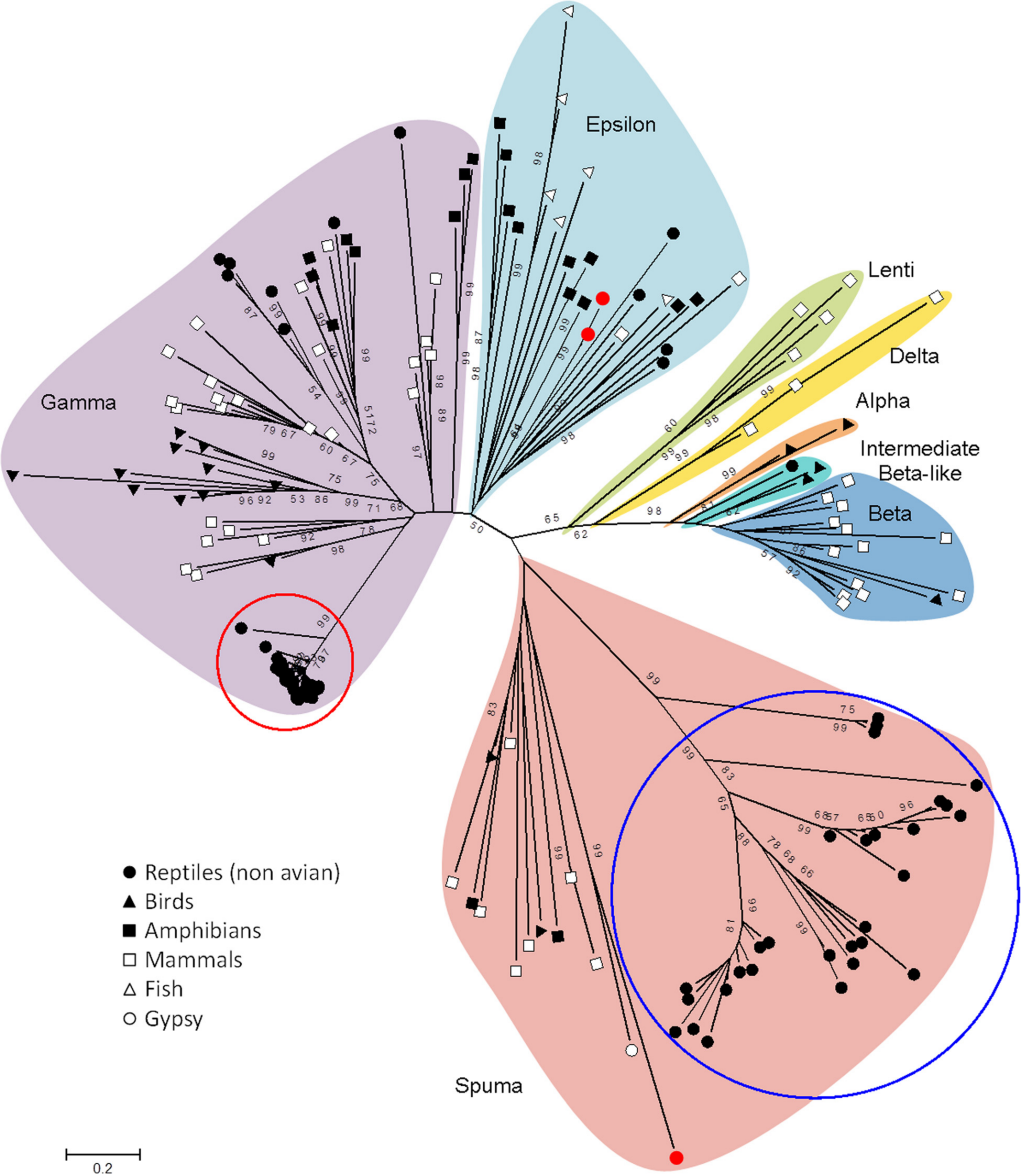
**Phylogenetic clustering of crocodilian ERVs (CERVs).** Neighbor joining tree based on aligned amino acid sequences from the retroviral *pro*-*pol* gene region. The final alignment length was 799 characters including gaps. Names near the shaded regions indicate the retroviral genus to which these sequences belong. The general host species taxa are indicated by symbols. The two major clades of ERVs found in crocodilians are indicated by colored circles: CERV1 in red and CERV2 in blue. Red dots indicate additional crocodilian ERV sequences. Numbers near branches indicate bootstrap support values.

Neighbor joining and maximum likelihood analyses incorporating retroviral sequences from non-crocodilian taxa consistently placed the CERV1 sequences with the *Gammaretrovirus* related ERVs. CERV2 related sequences consistently clustered with the *Spumaviruses*. Haplotype 58 clustered with the *Epsilonretrovirus* related ERVs, while haplotype 119 was placed within the *Spumaviruses* but separate from the CERV2 sequences (Figure 2). While there appears to be no host species-related sorting among CERV1 sequences, groupings within CERV2 suggest some degree of lineage specific evolution at the level of host family. A *Crocodylidae* specific group was observed, consisting of sequences from *C*. *porosus*, *C*. *niloticus*, and *C*. *mindorensis* (the Philippine crocodile). Haplotype 107 was placed midway between this lineage and the majority of known CERV2 sequences, which consist mostly of those isolated from the alligators and caimans. Notably, within clade CERV2, the majority of sequences from *Crocodylidae* cluster together, while those from *Alligatoridae* appear to be more divergent from each other.

## Discussion

The data presented in this study suggest that there are high levels of sequence diversity in *C*. *porosus* ERV sequences. The sequences isolated in this study correspond largely with the two major CERV clades previously identified. In addition, we have identified novel sequences that appear to be related to other retroviral genera. Within the clades, we have found evidence of strong purifying selection acting across both of the major clades, which is suggestive of recent integration or transposition. We have found preliminary evidence to propose the presence of sublineages within clade CERV2. While it is unlikely that this study encompasses the full extent of retroviral diversity in *C*. *porosus*, the data generated here provides a comprehensive insight into the process by which ERVs have populated the genomes of crocodilians, and their evolution within the genome of this species.

### High diversity present in CERV clades

A large number of novel sequences were generated, with very few haplotypes being recovered more than once. This high diversity of ERVs haplotypes within *C*. *porosus* can be explained by several possible scenarios: i) several recent and independent infection events by exogenous retroviruses from the various retroviral genera that have resulted in the current ERV diversity; ii) a single infection event by an exogenous retrovirus from each of the represented retroviral genera followed by repeated re-infection by the same ERV lineages; iii) a single infection event by an exogenous retrovirus from each of the represented retroviral genera followed by replication of ERVs within the genome, either by retrotransposition or complementation.

The similarity of sequences within each of the two major clades within *C*. *porosus* would make the first of these scenarios unlikely. Of the remaining scenarios, the second scenario would appear to explain the pattern of evolution seen in clade CERV1, while the presence of shared stop codons in CERV2 suggests proliferation through retrotransposition or complementation [4]. Further support for each of these methods of replication will be discussed in the following sections. Both of these scenarios result in many lineages of related retroviruses that can replicate and mutate independently [17]. This leads to a collection of proviruses that show high levels of nucleotide and amino acid diversity while at the same time retaining the original sequence characteristics, as seen in this study. This level of sequence diversity is not uncommon for ERVs, and is comparable to what has been observed in mammalian ERVs [31,32].

A low frequency of recombinant sequences was detected among the crocodilian ERV sequences, suggesting that recombination does not play a large role in the generation of ERV diversity in *C*. *porosus*. The detection of recombinant sequences where the predicted parental sequences were isolated from other species is indicative of ancestral recombination events. Given the rarity of cross species transmission [13] and the distinct distributions of crocodilian species, it is unlikely that these lineages arose from cross species transmission of ERVs between crocodilian species.

### Potential functionality of CERV clades

It is plausible that CERV1 may still be active within the genome of *C*. *porosus*, replicating through re-infection of host cells. Re-infection restores the fitness of the replicating ERV, thereby increasing preservation of the lineage [9,18] and allowing for further proliferation within the host genome. Based on the high number of sequence variants and high level of sequence similarity, we propose that this clade represents the product of a fairly recent integration event. The majority of stop codons present within this clade occupied unique positions within each sequence, indicating that it is unlikely that these ERVs arose through retrotransposition or by complementation with other related ERVs [4]. This is further supported by the presence of sequences with intact ORFs, and the strong purifying selection that has been observed within this clade [9,18].

The observed clustering of sequences encoding intact ORFs suggests that there may be a particularly active strain of ERV that has largely managed to escape inactivation within *C*. *porosus*. This clade also includes a number of available sequences from other species within *Crocodylidae*, suggesting that there may also be active ERVs present in these species. While most ERV insertions are inactivated by mutation shortly after integration, it is plausible that active lineages may still retain their capacity to replicate by re-infection well after species divergence [33,34].

On the other hand, shared stop codons in sequences from clade CERV2 mean that replication by re-infection is unlikely, lending support to retrotransposition or complementation as possible means of replication. Replication by either of these methods does not require that all genes are functional. Retrotransposition, for example, does not require a functional *env* domain [9,18]. Complementation on the other hand does not require functional proteins within the provirus, providing that the required regulatory regions within the long terminal repeats (LTRs) are intact, and that missing functional proteins are supplied by an exogenous retrovirus or partially intact ERV [4,18]. The strong levels of purifying selection detected in this clade suggest that this clade has recently been active, although sequence data from the other retroviral domains are needed to determine the likely method of replication.

### Low levels of phylogenetic resolution

The rapid proliferation of ERVs within a host genome can also confound attempts to differentiate ERVs by phylogenetic analyses. This is especially in the case of recent integration events where not enough evolutionary time has passed to allow insertions to develop distinguishing or phylogenetically informative mutations. In addition, the mutation rates of the *propol* domain are, comparatively, the lowest of the various retroviral domains [35]. While this characteristic makes this region ideal for studies of ERV proliferation across taxa, it could be argued that regions with typically higher mutation rates such as *gag* or *env* may be more appropriate for generating phylogenies within a species [19,35].

Furthermore, studies into the nucleotide substitution rate of crocodilian nuclear and mitochondrial sequences suggest that this is much lower in crocodilians than in most other vertebrates [24-27]. Thus, degenerate ERV sequences are likely to accumulate changes at a slower rate in crocodilians than most other vertebrate species, leading to a low level of host lineage specific evolution, as well as low levels of lineage differentiation. This has the effect of reducing our ability to detect host species specific lineages based on this data alone.

For this reason, it could also be argued that other more quickly evolving retroviral domains should be considered to provide resolution between host specific lineages. The characterization of the remaining ERV domains will also provide further insights into the methods of replication, and potential for re-infection. As such, future studies into the diversity of ERVs within crocodilians should now be oriented towards characterizing the entire length of proviral insertions rather than individual domains.

### Estimated infection times of the ERV clades

Strong purifying selection on both ERV clades suggests a recent population expansion may have occurred. Regardless of the method by which this is achieved, replication of elements results in the expansion of the population and the creation of autonomous, but related lineages that are capable of replicating and evolving independently [17]. In relatively recent population expansions, therefore, it would be expected that sequences would still share high levels of sequence similarity. This is corroborated by short internal branch lengths and the lack of phylogenetic resolution seen across the CERV alignments.

In the absence of LTRs or knowledge of the founding retroviral sequence, the ages of the initial integration events of the crocodilian ERVs can only be estimated from what is known about the species phylogenies and the assumed presence or absence of the ERVs in each of these species. Based on nucleotide and amino acid similarity to previously classified ERVs, and the presence of conserved retroviral motifs, we propose that the ERV complement of *C*. *porosus* came about through infection by three related lineages of retroviruses belonging to the gamma-, epsilon-, and spumaviral genera. The first of these infections would be that leading to the CERV2 clade, as sequences have been identified in species representing all families within Crocodylia. This integration is likely to pre-date the Alligator-Crocodile split, approximately 90 million years ago (MYA) [36]. The infection that gave rise to the CERV1 clade is likely to have occurred after this time period. The presence of nearly identical CERV1 sequences in other species within *Crocodylidae* would indicate that this integration could have occurred prior to diversification of the various crocodile species, at least 20 to 30 MYA [36].

Sequence divergence and phylogenetic evidence supports the presence of three sublineages within CERV2. These three groups are characterized by a number of diagnostic positions including shared frameshift mutations and stop codons within each group, and is moderately supported by bootstrap analyses on the phylogenetic trees of the CERV2 clade. This evidence furthers the notion that this clade represents an older integration event that has been present in the genome for a sufficient amount of time for the differentiation of distinct sequence lineages.

In the case of the *Epsilonretrovirus* related sequence, haplotype 58, the full extent of its proliferation within crocodilians is not known, as only one other similar sequence has been isolated - from *Gavialis gangeticus*, the gharial [9]. Thus, it cannot yet be determined whether this lineage is also present among crocodilians, or if it is the result of cross species retroviral infection involving a limited number of crocodilian species. The apparent rarity of this sequence within the genome also raises questions as to why it has not multiplied further, or why it is so much less likely to be detected. Deeper or different sampling strategies may be required to understand the reasons behind this.

### Reclassifying CERV2

Contrary to the data presented by Jaratlerdsiri *et al.*[11], CERV2 related sequences were grouped within the *Spumaviruses* rather than forming a separate distinct clade. This can mainly be attributed to the use of different alignment algorithms between studies. The conventional strategy of high gap penalties within global alignments can be problematic when aligning highly divergent sequences, such as ERV sequences, where the use of high gap penalties can result in the forced alignment of non-homologous sequence regions [37]. Instead, we elected to use the alignment program MAFFT, which implements algorithms specifically designed for the alignment of highly divergent sequences. These algorithms result in alignments based around a series of local alignments of conserved regions, and allows for long lengths of un-alignable sequence between the conserved domains [38]. In studies such as this, where ERV discovery is, more or less, *de novo*, we believe that this may be a more effective method for sequence comparison, as novel sequences may share only a low level of similarity with known sequences, making them difficult to align and potentially reducing the power of downstream analyses.

## Conclusions

We propose that the ERV complement of *C*. *porosus* has come about through a combination of recent infections and replication of ancestral ERVs. Two major clades are present as a result of infection by gammaretroviral and spumaviral lineages. Strong purifying selection acting on these clades suggests that this activity is recent or still occurring in the genome of this species. We have uncovered a large amount of sequence variation within both of the major clades of ERV present in *C*. *porosus*, as well as the presence of an additional lineage that appears to be present in the genome to a much lesser degree. While no host taxa dependent clustering was observed, there is evidence for the divergence of sublineages within the more ancient ERVs in *C*. *porosus*. The discovery of potentially functional elements is an interesting development that warrants further investigation.

## Methods

### Sampling

Blood samples were collected from *C*. *porosus* hatchlings from nests across 17 locations, representing nine river basins in the Northern Territory, Australia (Figure 1). The animals sampled were from eggs collected under the Northern Territory Government’s ranching program. One to two individuals per clutch were sampled, from a total of 45 clutches. Blood samples were collected from the cervical sinus as described by Lloyd and Morris [39]. DNA was extracted using the QIAamp DNA Mini kit (QIAGEN, Germantown, MD, USA).

### PCR amplification and sequencing

PCR was used to amplify a 700 to 1,000 bp region of the retroviral *propol* gene region using universal primers [40]. Amplicons were gel purified and cloned using the pGEM-T Easy Vector and JM109 *E*. *coli* cells (Promega, Madison, WI, USA) according to the manufacturer’s instructions. To ensure that the correct inserts were present, clones were verified by PCR, as described above, and by *EcoRI* enzyme digests after purification. Positive clones were purified and sequenced using Sanger sequencing at the Australian Genome Research Facility (AGRF; Brisbane, QLD, Australia).

### Sequence alignment and analysis

Nucleotide sequences were aligned using CLUSTALW [41] as implemented in the program package MEGA5 [42]. Representatives of the major sequence groups identified here were compared with previously identified ERV sequences in the GenBank and RepBase databases using BlastX [43] and Censor [44] respectively. Unique haplotypes from this study were identified using FaBox [45] and re-aligned against other similar sequences generated in this study using the program MACSE [46]. The resulting alignments were translated in MEGA5 [42] using the standard vertebrate genetic code tables, and putative amino acid sequences were aligned in CLUSTALW using the BLOSUM matrix with residue specific and hydrophilic penalties, and high gap penalties as described by Xiong and Eickbush [47]. The presence of conserved retroviral motifs and domains was assessed based on similarity to motifs defined by Sperber *et al.*[48]. Genetic distances were calculated using the Jukes-Cantor model for the nucleotide alignments and the JTT model for amino acid alignments. The presence of recombinant sequences was evaluated using the program RDP3 [49] with default program settings.

### Phylogenetic analysis

Phylogenetic analyses were used to detect evidence of sublineages within each of the major clades. For both major clades, neighbor joining and maximum likelihood analyses were carried out with 1,000 bootstrap replicates and representative sequences from the respective retroviral genera as outgroups (HERV-E for CERV1 and HERV-L for CERV2). Neighbor joining trees were created in MEGA5 [42] using the Jukes-Cantor and Poisson corrections to account for multiple substitutions. The best fit model of substitution (CERV1: HKY, JTT; CERV2: GTR, JTT for nucleotide and amino acids respectively) was determined using Model Generator [50] and implemented in PhyML [51].

Additional phylogenetic analyses were performed to assess the evolutionary relationship of the novel *C*. *porosus* sequences with other published ERV sequences. This data set comprised of five representative novel sequences from this study, 55 published sequences from other species within Crocodylia and 113 published sequences from other species [9,11,13,14,19]. Due to the highly diverse nature of the sequences from the various species, sequences were aligned using the program MAFFT and the E-INS-i algorithm [38]. Phylogenetic trees were created as described above.

### Tests for selection

Codon based Z-tests were carried out in MEGA5 [42] to investigate overall selective forces acting on the two major CERV clades in *C*. *porosus*. Data sets were analyzed using the Nei and Gojobori method with the Jukes-Cantor correction to account for multiple substitutions [52]. Tests were conducted to test for non-neutrality, positive and purifying selection. Synonymous and non-synonymous ratios were also calculated for these data sets in PAML v4.4 [53] using a likelihood ratio test (LRT) to assess significance of the detected selection signatures.

Further details on the amplification conditions, RDP program settings, selection criteria for representative sequences and the PAML model comparisons are available in Additional file 2.

## Abbreviations

CERV: Crocodilian endogenous retrovirus; ERV: Endogenous retrovirus; LRT: Likelihood ratio test; LTRs: Long terminal repeats; MYA: Million years ago; ORF: Open reading frame; PCR: Polymerase chain reaction; RIRDC: Rural Industries Research and Development Corporation.

## Competing interests

The authors declare that they have no competing interests.

## Authors’ contributions

AYC carried out the molecular genetic studies and drafted the manuscript as part of her doctoral studies. SJA contributed with some molecular studies and assisted with early drafts of the manuscript. SI collected the samples used in this study and helped draft the manuscript. JG conceived, designed and supervised this study and helped draft the manuscript. All authors read and approved the final manuscript.

## Supplementary Material

Additional file 1 **Supplementary Figures and Tables.** Contains Supplementary Figures 1 and 2, and Supplementary Tables 1 and 2. Click here for file

Additional file 2 **Further details on methods.** Contains further details on the amplification conditions, RDP program settings, selection criteria for representative sequences, and the PAML model comparisons [54-60]. Click here for file
