# Supplementary material for: Strong purifying selection in endogenous retroviruses in the saltwater crocodile (Crocodylus porosus) in the Northern Territory of Australia
Source: Mob DNA. 2012 Dec 5;3:20. doi: 10.1186/1759-8753-3-20 (PMC3531266; doi:10.1186/1759-8753-3-20)
Supplement: Additional file 1 — Supplementary Figures and Tables. Contains Supplementary Figures 1 and 2, and Supplementary Tables 1 and 2. [file 1759-8753-3-20-S1.pdf]

Strong purifying selection in endogenous retroviruses in the saltwater crocodile (*Crocodylus porosus*) in the Northern Territory of Australia: Additional File 1

This file contains:

Supplementary Figure 1

Supplementary Figure 2

Supplementary Table 1

Supplementary Table 2

Supplementary Figures

(a)

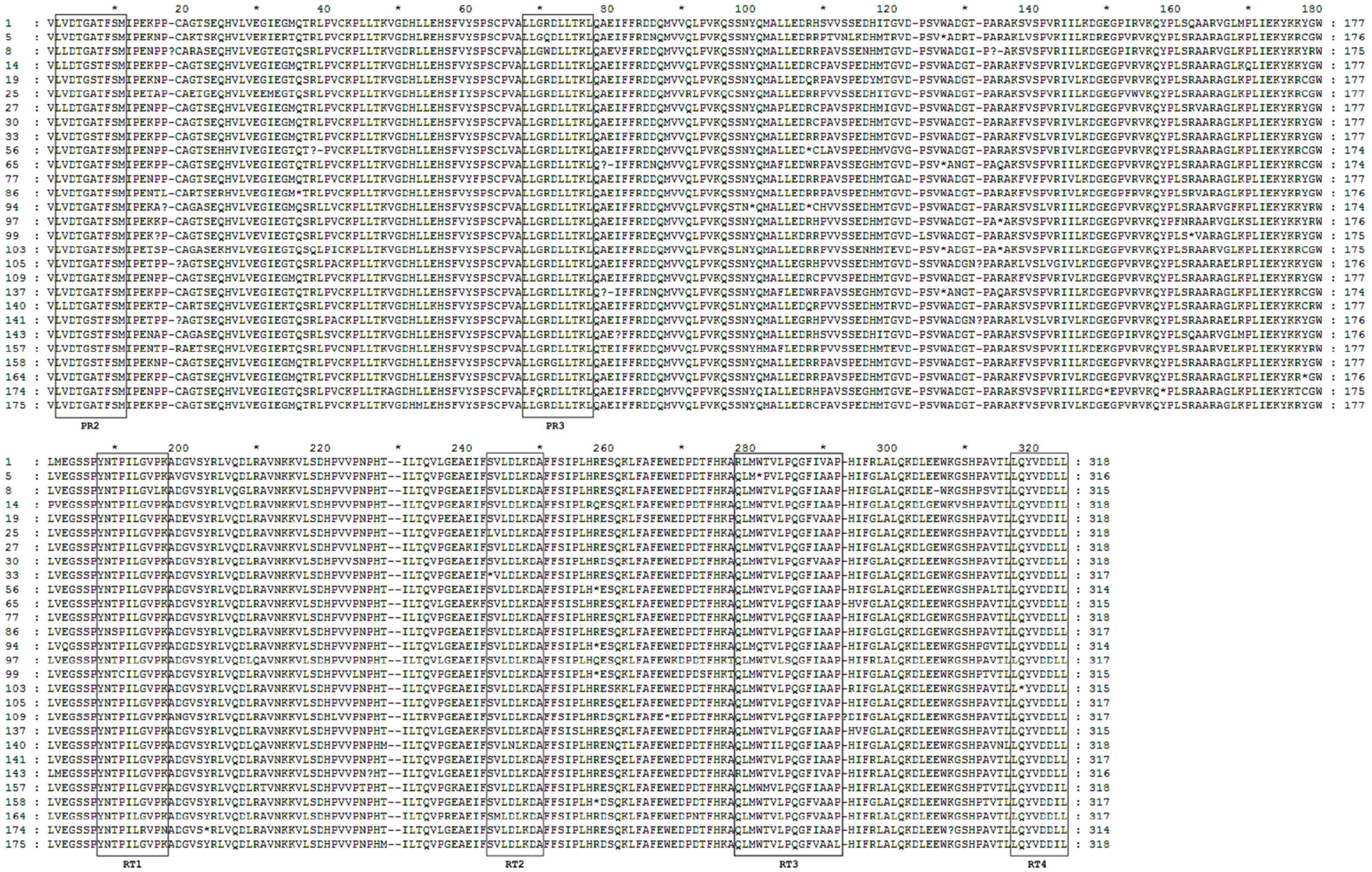

(b)

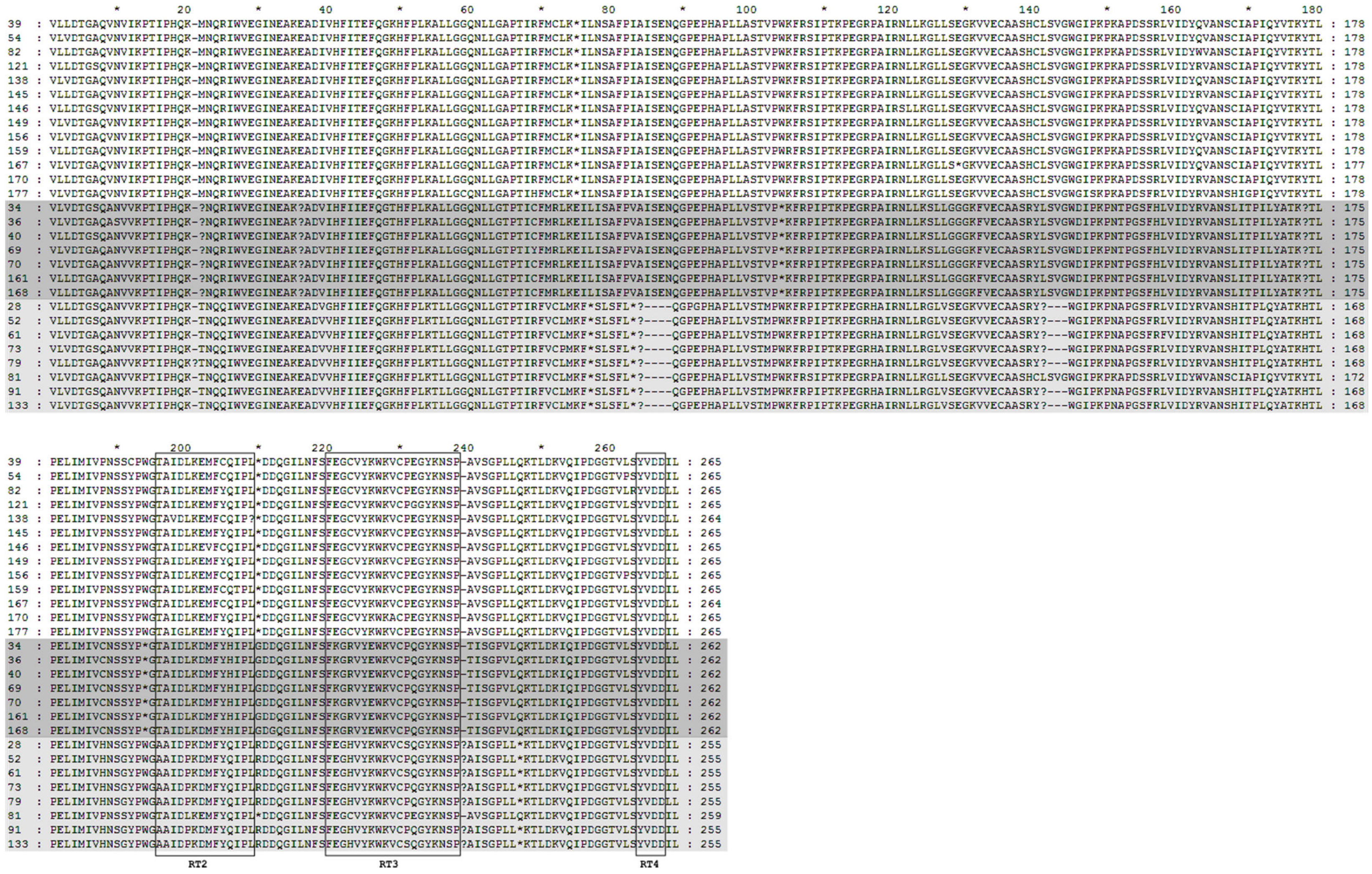

Supplementary Figure 1: Alignments of putative amino acid translations of selected sequences from clades CERV1 (a) and CERV2 (b). Conserved retroviral motifs are bound by black boxes. The dark and light shaded boxes in (b) highlight the proposed sub-lineages within clade CERV2: the unshaded sequences are group CERV2a, dark grey are CERV2b, and lighter grey are CERV2c.

(a)

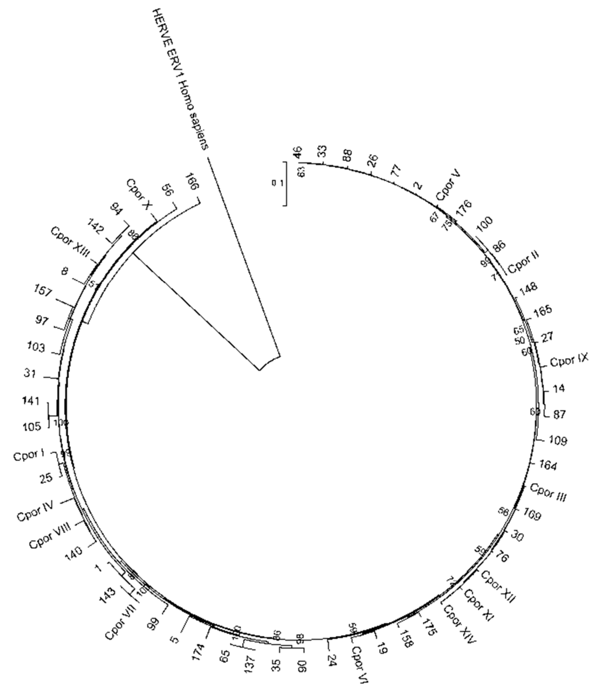

(b)

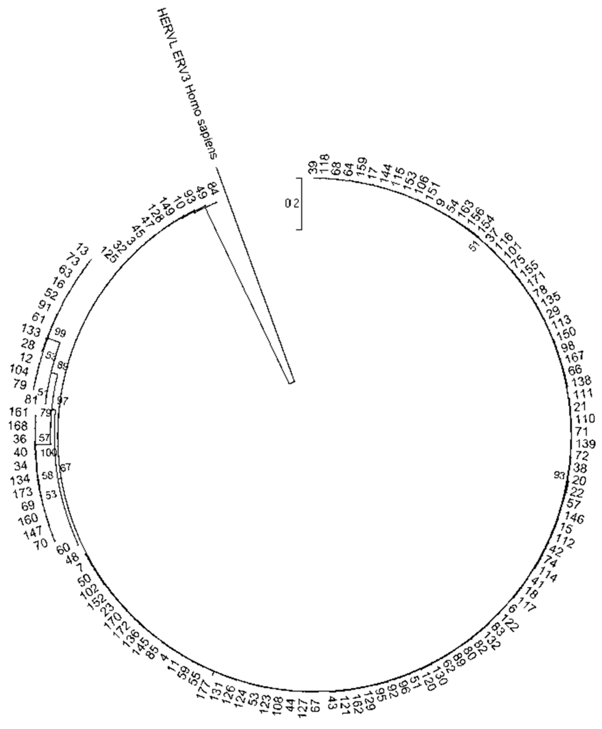

Supplementary Figure 2: Neighbour joining trees based on the nucleotide alignments of *C. porosus* sequences from the CERV1 (a) and CERV2 (b) clades. Outgroups are human ERV sequences extracted from the Repbase database and from the same general ERV classes as these sequences. Numbers outside the circles indicate the haplotypes included in each tree. Smaller numbers within the circle represent bootstrap support values greater than 50%.

Supplementary Tables

Supplementary Table 1: Predicted recombinant and parental sequences as determined by RDP

Recombinant Sequence	Breakpoint Positions Begin	Breakpoint Positions End	Major Parental Sequence[a]	Minor Parental Sequence[b]	Probability that the detected sequence is a recombinant[c] RDP	GENECONV	Bootscan	Maxchi	Chimaera	SiSscan	3Seq
CERV1-All											
1	289	906	148	Cpor_VII	0.0230	NS	0.0234	0.0001	0.0107	0.0000	0.0000
Csia_IV	753	872	164	142	0.0301	NS	0.0306	NS	NS	NS	0.0344
Cnil_I			164	142	Trace evidence only						
CERV1-porosus											
1	289	869	175	Cpor_VII	0.0005	NS	0.0005	0.0001	0.0021	NS	0.0000
CERV2-All											
81	23	296	53	52	0.0001	0.0020	0.0001	0.0001	0.0001	0.0000	0.0000
Amis_II	698	1	Asin_IV	Ppal_II	0.0398	NS	NS	NS	NS	NS	0.0215
Amis_I			Asin_IV	Ppal_II	Trace evidence only						
CERV2-porosus											
81	23	296	53	52	0.0001	0.0013	0.0001	0.0001	0.0001	0.0000	0.0000
60	16	191	162	34	0.0329	NS	NS	NS	NS	NS	0.0003

[a] Major Parent: Parent contributing the larger fraction of sequence
[b] Minor Parent: Parent contributing the smaller fraction of sequence
[c] NS: No significant P-value was recorded for this recombination event using this method

Supplementary Table 2: PAML analysis showing parameter estimates, average d_N/d_S, and significance of the pairwise test comparisons

H_n	Model	Average d_N/d_S	PAML parameter estimates	lnL	LRT test statistic	*df*	p value
**CERV1**							
H0	M0: One ratio	0.4904	ω = 0.49042	-6936.0525	273.3946	4	< 0.001
H1	M3: Discrete	0.5187	p0 = 0.63002 p1 = 0.36034 p2 = 0.00964<br>ω0 = 0.14883 ω1 = 0.97228 ω2 = 7.73167	-6799.3552			
H2	M1a: Nearly neutral	0.4344	p0 = 0.66860 p1 = 0.33140P2<br>ω0 = 0.15403 ω1 = 1.00000	-6845.3939	92.0213	2	< 0.001
H3	M2a: Positive selection	0.5234	p0 = 0.64094 p1 = 0.34945 p2 = 0.00962<br>ω0 = 0.15461 ω1 = 1.00000 ω2 = 7.78576	-6799.3832			
H4	M7: Beta	0.4217	p = 0.37192 q = 0.50996	-6848.2309	96.8512	2	< 0.001
H5	M8: Beta & ω	0.4973	p0 = 0.99019 p = 0.40522 q = 0.54097<br>(p1 = 0.00981) ω = 7.46662	-6799.8053			
**CERV2**							
H0	M0: One ratio	0.5539	ω = 0.55393	-4029.5600	413.1006	4	< 0.001
H1	M3: Discrete	0.6898	p0 = 0.79438 p1 = 0.18634 p2 = 0.01928<br>ω0 = 0.14019 ω1 = 1.66189 ω2 = 13.93868	-3823.0097			
H2	M1a: Nearly neutral	0.3057	p0 = 0.76252 p1 = 0.23748<br>ω0 = 0.08951 ω1 = 1.00000	-3921.6374	178.2283	2	< 0.001
H3	M2a: Positive selection	0.5864	p0 = 0.72223 p1 = 0.25559 p2 = 0.02218<br>ω0 = 0.10433 ω1 = 1.00000 ω2 = 11.51932	-3832.5232			
H4	M7: Beta	0.3349	p = 0.17755 q = 0.35268	-3923.0792	164.9022	2	< 0.001
H5	M8: Beta & ω	0.6217	p0 = 0.97733 p = 0.21801 q = 0.39323<br>(p1 = 0.02267) ω = 12.04341	-3840.6281			
