# Supplementary material for: Strong purifying selection in endogenous retroviruses in the saltwater crocodile (Crocodylus porosus) in the Northern Territory of Australia
Source: Mob DNA. 2012 Dec 5;3:20. doi: 10.1186/1759-8753-3-20 (PMC3531266; doi:10.1186/1759-8753-3-20)
Supplement: Additional file 2 — Further details on methods. Contains further details on the amplification conditions, RDP program settings, selection criteria for representative sequences, and the PAML model comparisons [54-60]. [file 1759-8753-3-20-S2.docx]

# Strong purifying selection in endogenous retroviruses in the saltwater crocodile (*Crocodylus porosus*) in the Northern Territory of Australia: Additional File 2

This file contains:

PCR conditions

RDP settings

Selection criteria for representative sequences

PAML model comparisons

**PCR conditions**

Forward primer sequence: GTK TTI KTI GAY ACI GGI KC

Reverse primer sequence: ATI AGI AKR TCR TCI ACR TA

PCR was carried out in duplicate, in 25 µL reaction volumes, containing 100pmol of each primer, 2mM MgCl_2_, 0.16mM dNTPs, PCR buffer and 1U of *Taq* polymerase. PCR cycles were as follows: initial denaturation at 94°C for 2 minutes, 35 cycles of 45°C (30 seconds), 72°C (60 seconds) and 94°C (30 seconds), followed by final annealing period of 3 minutes at 45°C and a final extension period of 10 minutes at 72°C.

**RDP settings**

Default program settings were used, implementing the RDP [1], GENECONV [2], Bootscan [3], MaxChi [4], Chimaera [5], SiScan [6] and 3seq methods [7] for detection of recombinants with a significance cut off of p = 0.05 and the Bonferroni correction. Tests were carried out on sequences within *C. porosus* and across species. Sequences were considered to be potential recombinants if two or more of the above methods returned a significant value.

**Selection criteria for representative sequences**

Due to the large number of sequences recovered, representative sequences from *C. porosus* selected based on similarity to a consensus sequence from each clade. Since we are also interested in the functionality of sequences, sequences with fewer perceived indels or stop codons were selected over those with more of these mutations where there were multiple equally similar sequences. Sequences from other crocodilians were treated similarly except in cases where there were clearly two divergent lineages represented.

**PAML model comparisons**

Tests for selection on specific sites were conducted under the assumption of a single rate of substitution across branches. The models M0 and M3 were compared to determine if selection differed between sites, and the model pairs M2 and M3, and M7 and M8 were used to test for selection at each site. Likelihood ratio test (LRT) statistics were calculated for the following pairs to determine significance; M0 – M3, M1a – M2a, M7 – M8 (see Results for additional information)

LRT values were calculated as twice the difference between the likelihood values for each of the different models, and compared to the Chi-squared values for one degree of freedom.

1. Martin D, Rybicki E: **RDP: detection of recombination amongst aligned sequences.** *Bioinformatics* 2000, **16:**562-563.

2. Padidam M, Sawyer S, Fauquet CM: **Possible emergence of new geminiviruses by frequent recombination.** *Virology* 1999, **265:**218-225.

3. Martin DP, Posada D, Crandall KA, Williamson C: **A modified bootscan algorithm for automated identification of recombinant sequences and recombination breakpoints.** *AIDS Res Hum Retroviruses* 2005, **21:**98-102.

4. Smith JM: **Analyzing the mosaic structure of genes.** *J Mol Evol* 1992, **34:**126-129.

5. Posada D, Crandall KA: **Evaluation of methods for detecting recombination from DNA sequences: Computer simulations.** *Proc Natl Acad Sci U S A* 2001, **98:**13757-13762.

6. Gibbs MJ, Armstrong JS, Gibbs AJ: **Sister-scanning: a Monte Carlo procedure for assessing signals in recombinant sequences.** *Bioinformatics* 2000, **16:**573-582.

7. Boni MF, Posada D, Feldman MW: **An exact nonparametric method for inferring mosaic structure in sequence triplets.** *Genetics* 2007, **176:**1035-1047.
